# Accelerating the acquisition of the 3D Dual Cardiac Phase technique using RPE trajectories

**DOI:** 10.1186/1532-429X-16-S1-W38

**Published:** 2014-01-16

**Authors:** Karis Letelier, Marcelo E Andia, Cristian Tejos, Pablo Irarrazaval, Claudia Prieto, Sergio Uribe

**Affiliations:** 1 Biomedical Imaging Center, Pontificia Universidad Católica de Chile, Santiago, Chile; 2 Electrical Engineering Department, Faculty of Engineering, Pontificia Universidad Católica de Chile, Santiago, Chile; 3 Radiology Department, School of Medicine, Pontificia Universidad Católica de Chile, Santiago, Chile; 4 Division of Imaging Sciences and Biomedical Engineering, Kings College London, London, London, UK

## Background

A 3D Dual Cardiac Phase (3D-DCP) scan was proposed to obtain systolic and diastolic images with equivalent quality and scan time compared to the 3D single cardiac phase acquisition (Uribe et al, Radiology 2008). In this work, we propose to accelerate the acquisition and reconstruction of the 3D-DCP approach by sharing information from the outer k-space of both cardiac phases using Radial Phase Encoding (RPE) trajectories (Boubertak, et al., MRM 2009) and gridding-CLEAR reconstruction

## Methods

The RPE trajectory was implemented in a 1.5T Philips clinical scanner. The acquisition scheme for the diastolic phase was shifted with respect to the systolic phase. In five volunteers, a fully-sampled 3D-DCP scan was acquired using a 5-channel coils to determine the percentage of k-space that can be shared between both cardiac phases. Thereafter, undersampled 3D-DCP data with undersampling factors of 2, 4 and 8 were acquired in 10 volunteers. The fully-sampled data were retrospectively undersampled and different percentages of the outer k-space were shared between both phases. The Root Means Square Error (RMSE) was calculated between the fully-sampled image and the images reconstructed using the sharing approach. From the RMSE curve, we determined a specific percentage of the two phases that was used to reconstruct the undersampled data sets. The images were reconstructed using iterative SENSE (eight iterations) and gridding with uniform signal combination of the coils (CLEAR) for undersampling factors of 2 and 4. Finally, two experienced users performed an image quality assessment and a cardiac volume analysis.

## Results

**Figure 1:**
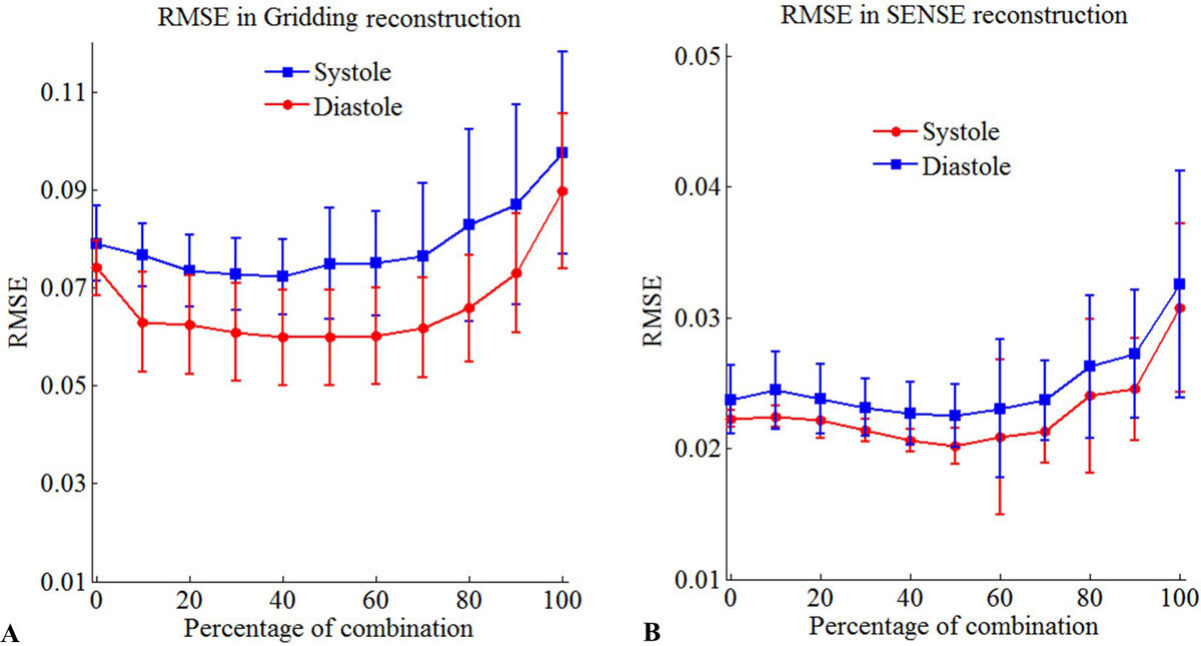
**RMSE between the images acquired with 144 phase encoding (RPE data no undersampled) and 72 phase encoding combining different percentage of k-space information between the systolic and diastolic phases**. A) Gridding reconstruction. B) SENSE reconstruction.

**Figure 2:**
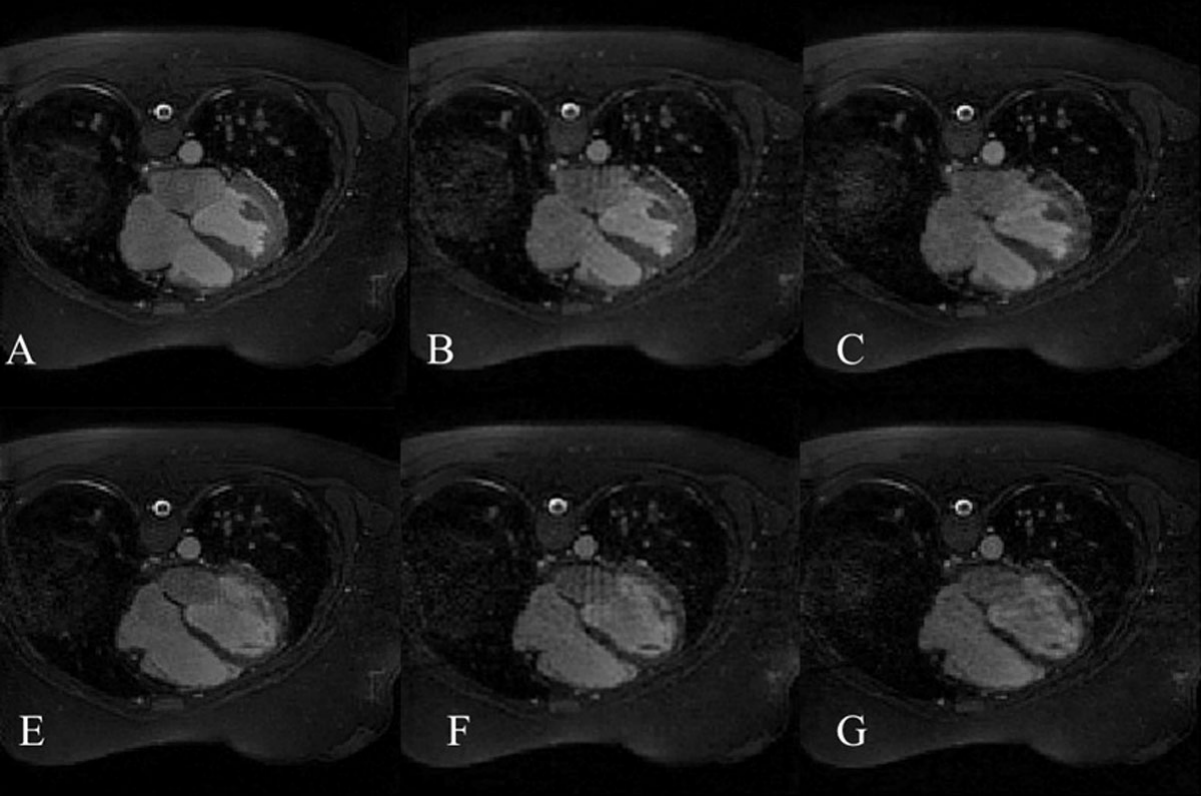
**End systolic (top row) and End diastolic (bottom row) images obtained in one volunteer**. Images obtained with iterative SENSE reconstruction are shown in A, E and B, F for undersampling factors of 2 and 4, respectively. In C and G, we show the images obtained with an undersampling factor of four by combining 50% of the information between both cardiac phases and reconstructing using the gridding-CLEAR approach. Negligible difference can be appreciated between the images reconstructed with gridding-CLEAR and the images reconstructed with iterative SENSE for the same undersampling factors.

Figure 1 shows how that the RMSE decreases when the percentage of shared information increases. It can be noted that when a 40-60% of combination is used, the RMSE is minimum. The reconstructed images are shown in Figure 2. Results of the image quality assessment showed that images obtained with undersampling factor of 4 and reconstructed with gridding-CLEAR using 50% phase combination are comparable to images reconstructed with iterative SENSE. Results of the cardiac volume analysis (not shown here) indicate no statistical differences between volumes calculated from the images obtained using the sharing strategy with an undersampling factor of 4 and the images reconstructed using iterative SENSE with an undersampling factor of 2. In addition, the proposed approach reduces the reconstruction time from 40 minutes to 60 seconds.

## Conclusions

We have proposed a method that obtains 3D-DCP scans with an image quality equivalent to those reconstructed with iterative SENSE, but within a clinically acceptable reconstruction time.

## Funding

Anillo ACT 079 and FONDECYT #11100427.

